# Identification of small molecule enzyme inhibitors as broad-spectrum anthelmintics

**DOI:** 10.1038/s41598-019-45548-7

**Published:** 2019-06-24

**Authors:** Rahul Tyagi, Mostafa A. Elfawal, Scott A. Wildman, Jon Helander, Christina A. Bulman, Judy Sakanari, Bruce A. Rosa, Paul J. Brindley, James W. Janetka, Raffi V. Aroian, Makedonka Mitreva

**Affiliations:** 1 McDonnell Genome Institute, Washington University School of Medicine, 4444 Forest Park Ave, St. Louis, Missouri 63108 USA; 2 University of Massachusetts Medical School, Suite 219 Biotech 2, 373 Plantation St., Worcester, Massachusetts 01605 USA; 3 UW Carbone Cancer Center, School of Medicine and Public Health, University of Wisconsin-Madison, 1111 Highland Ave., Madison, Wisconsin 53792 USA; 4 Department of Biochemistry and Molecular Biophysics, Washington University School of Medicine, 660S. Euclid Ave., Box 8231, St. Louis, Missouri 63110 USA; 5 Department of Pharmaceutical Chemistry, University of California San Francisco, 1700 4th St, San Francisco, California 94158 USA; 6 Department of Microbiology, Immunology & Tropical Medicine, and Research Center for Neglected Diseases of Poverty, School of Medicine and Health Sciences, George Washington University, Ross Hall, Room 521, 2300 I Street, NW, Washington, DC 20037 USA; 7 Division of Infectious Diseases, Department of Medicine, Washington University School of Medicine, 4523 Clayton Ave., CB 8051, St. Louis, MO 63110 USA

**Keywords:** Molecular medicine, Parasitic infection, Translational research, Phenotypic screening

## Abstract

Targeting chokepoint enzymes in metabolic pathways has led to new drugs for cancers, autoimmune disorders and infectious diseases. This is also a cornerstone approach for discovery and development of anthelmintics against nematode and flatworm parasites. Here, we performed omics-driven knowledge-based identification of chokepoint enzymes as anthelmintic targets. We prioritized 10 of 186 phylogenetically conserved chokepoint enzymes and undertook a target class repurposing approach to test and identify new small molecules with broad spectrum anthelmintic activity. First, we identified and tested 94 commercially available compounds using an *in vitro* phenotypic assay, and discovered 11 hits that inhibited nematode motility. Based on these findings, we performed chemogenomic screening and tested 32 additional compounds, identifying 6 more active hits. Overall, 6 intestinal (single-species), 5 potential pan-intestinal (whipworm and hookworm) and 6 pan-Phylum Nematoda (intestinal and filarial species) small molecule inhibitors were identified, including multiple azoles, Tadalafil and Torin-1. The active hit compounds targeted three different target classes in humans, which are involved in various pathways, including carbohydrate, amino acid and nucleotide metabolism. Last, using representative inhibitors from each target class, we demonstrated *in vivo* efficacy characterized by negative effects on parasite fecundity in hamsters infected with hookworms.

## Introduction

Metabolic potential is a critical determinant for a pathogen’s development and growth, infectivity and maintenance^1–5^. Consequently, the enzymes comprising the metabolic network of a pathogen are potential targets for drug discovery. In fact, multiple drugs in current clinical and agricultural use as anti-infective agents target these enzymes^2,3,5^. However, this approach has not been suitable for the discovery of anthelmintics until recently, due to insufficient knowledge regarding the metabolism of parasitic nematodes - partly due to large metabolic diversity within the phylum, especially amongst those inhabiting distinct trophic niches. Moreover, until recently, there has also been a lack of a comprehensive knowledgebase of their genomes and biochemistry. This newly generated genomics information^4,6^ significantly facilitates and expedites the development of drugs targeting specific parasites, and also enables comparative studies to infer shared similarities in diverse parasites that could be exploited to design anthelmintics with a broad-spectrum potential. Such anthelmintics are especially desirable because concomitant infections with different parasites are predominant in endemic countries^7–9^.

In an earlier study to identify novel broadly effective anthelmintics^1^, we followed an omics-driven target and drug prioritization approach to find potential metabolic enzyme drug targets using data from 10 Nematoda genomes, including four parasitic nematodes (a whipworm, a filarial worm, and two plant parasitic species). The premise of that work was that certain enzymes in the metabolic network form nodes that are especially vulnerable and hence are attractive candidates for drug-targeting. Specifically, targeting the enzymes that either uniquely produce or uniquely consume a substrate, called ‘chokepoint enzymes’^10^ is expected to harm the parasite by changing the concentration of the substrate to either too low or too high, potentially leading to morbidity or mortality. If carefully selected, employing insight into the biology and metabolic requirements of both the host and the pathogen, such targets have the potential to selectively harm the parasites without unacceptable side effects for the host. Based on the target and hit prioritization, seven compounds were selected and assayed *in vitro* in three highly divergent nematodes - *C*. *elegans*, a hookworm and a filarial species. Perhexiline, a putative inhibitor of carnitinepalmitoyl transferase (CPT) was effective against all three species. This work formed the basis of further exploration of putative CPT inhibitors repurposed for their anthelmintic potential^11^.

One limitation of the previous studies was the small number of nematode genomes available at the time, especially for parasitic worms. Moreover, even among the 10 genomes that were used, five belonged to the free-living genus *Caenorhabditis*. Fortunately the total number of nematode genomes and the diversity represented in those species has substantially increased in recent years, particularly with the publication of a large number of helminth genomes and their comparative analyses by International Helminth Genomes Consortium (IHGC^12^). Among the multiple analyses reported by IHGC was a comparison of the metabolic potential of nematodes, requiring us to develop a comprehensive method for enzyme annotation, and generate pathway reconstructions^13–15^, resulting in novel insights and confirmation of previous results that can be important for drug discovery and for understanding parasite biology and host-parasite interactions. The genomic data now available to us provide an opportunity to expand upon previous studies using a more representative set of parasitic worms for prioritizing chokepoint enzymes as drug targets and advancing the chemogenomic analysis, and phenotypic and *in vivo* assays.

Our primary objective was to identify drugs effective against whipworm and hookworm, which are among the most important human parasites. Moreover, we also aimed at covering ever broader phylogenetic and physiological range. Therefore, in this study, we strategically selected a set of 17 representative parasitic nematode species, spanning 4 of the 5 nematode phyla, including both intestinal and filarial worms, and used systems biology and evolutionary principles to reconstruct metabolic networks for these diverse parasites^11,13,15^. We were able to carry out more accurate gene- and single nucleotide-level comparative studies leveraging the recent advancements in genomic resources and metabolic databases. For instance, comparing a potential target gene among a diverse group of parasites and with the host gene sequence, critical sequence variations may be identified which are specific to the parasites, or are highly conserved among nematodes yet sufficiently divergent from the host^16^. Such omics-driven knowledge-based target prioritization approach^17,18^ followed by chemogenomic screening using large-scale drug databases (e.g. ChEMBL^19^) facilitated the identification of drug-like compounds with broad-spectrum control potential. We selected representative intestinal parasites at the extremes of the phylogeny^20^ (whipworm *Trichuris muris* from clade I of Nematoda and hookworm *Ancylostoma duodenale* from clade V) along with a phylogenetically distant lymphatic parasite (filarial *Brugia pahangi* from clade III) to conduct phenotypic screening of adult developmental stages of these worms to validate our predictions. The approach enabled the identification of inhibitors of key chokepoint enzymes that shared potentially pan-intestinal and pan-phylum efficacy. We also pursued iterative phenotypic screening guided by structure-activity relationships (SAR) to expand our list of lead compound candidates. The *in vivo* effect of representative compounds was also tested.

## Results and Discussion

### Identifying and prioritizing the metabolic enzyme chokepoints in parasitic nematodes

**Figure 1:**
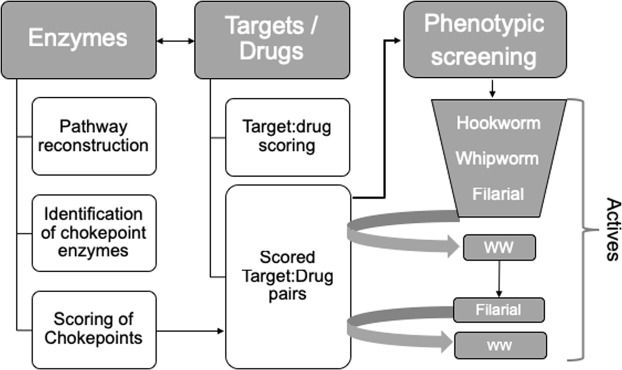
Flowchart outlining the overall analysis pipeline. ww = ”whipworm”.

The overall analysis process is outlined in Fig. 1 (for more details see Supplementary Fig. S1). A total of 669 unique ECs (Enzyme Commission IDs) were identified across 17 nematode species. Analysis of individually reconstructed metabolic networks led to identification of 389 ECs that were chokepoints in at least one species (Supplementary Table S1). Among these, 186 were taxonomically conserved^20^ across at least *Trichuris muris* (Clade I), *Ancylostoma ceylanicum* (Clade V) (the two species used for screening – “target species”) and at least one other Clade III or Clade IVa species (to ensure broad nematode conservation).

These 186 chokepoint ECs were ranked based on a weighted scoring method (Supplementary Fig. S1; Methods) which included phylogenetic conservation, number of metabolic pathways they are involved in, RNAi phenotype of the *C*. *elegans* ortholog, previous identification in literature, and RNAseq based expression levels in especially relevant developmental stage(s). Every nematode chokepoint from the two target species - intestinal nematodes representing the phylogenetic extremes of the phylum Nematoda (clade I, the whipworm *T*. *muris* and clade V, the hookworm *A*. *ceylanicum*) - was linked to ‘drug-like’ compounds in the ChEMBL^19^ database based on similarity to ChEMBL targets, resulting in 448 and 606 unique ChEMBL targets corresponding to 664 *A*. *ceylanicum* proteins and 756 *T*. *muris* proteins, respectively. Using pChEMBL^19^ values a total of 188,454 target:compound pairs were identified (pChEMBL score ≥5, corresponding to 10 μM IC_50_ etc.). Of these, 83,134 pairs included both a gene with an EC assignment and a compound with a “Quantitative Estimate of Druglikeness” (weighted QED) score recorded in the database. Intersection of these with the 186 selected nematode chokepoint ECs resulted in 22,498 conserved chokepoint ChEMBL target:compound pairs (82 targets/64 ECs paired to 12,395 unique compounds). The final ChEMBL target:compound scores were calculated, prioritizing target:chokepoint pairs that have high drug likeness and high target affinity. Multiplying the final nematode chokepoint EC score and the final ChEMBL target:compound score (Supplementary Fig. S1C) resulted in the final prioritization score. Thereafter, the 22,498 scored compound:target pairs (64 ECs) were reduced to 11,869 pairs (50 ECs) with a PDB^21^ (Protein Data Bank) structural match to the target. In the final step, the top 3 target:compound pairs per EC were selected (142 total target:compound pairs, 128 compounds; Supplementary Fig. S1D, Supplementary Tables S2, S3), and these were ranked according to the final prioritization score.

Out of the top 50 ECs, 10 ECs were prioritized based on associated compound availability and guided by literature review of the EC functions and potential for biological efficacy (Table 1). These ECs were categorized into five classes. The first class of target ECs was dehydrogenases, and included (i) L-lactate dehydrogenase (LDH; 1.1.1.27), previously suggested to be a potential anthelmintic target in *Clonorchis sinensis*^22^ and *Taenia asiatica*^23^. A wide variety of existing effective anthelmintics including Praziquantel, Levamisole, and Mebendazole have been found to result in LDH inhibition in trematode family Paramphistomidae^24^; (ii) Malate dehydrogenase (MDH; 1.1.1.37), inhibited by several major benzimidazole anthelmintics including Albendazole, although it is not their primary target^25^; (iii) Aldehyde dehydrogenase (NAD+) (ALDH; 1.2.1.3), previously explored as a potential target due to its impact on alcohol tolerance and cancer chemotherapy resistance. ALDH may be related to broad based drug resistance, and its inhibition might facilitate efficacy of other drugs^26^ and (iv) Dihydroorotate dehydrogenase (quinone) (DHODH; 1.3.5.2), studied as a target for protozoan (malaria and trypanosome) parasites^27–30^. The second class of EC targets was kinases, and included (i) Hexokinase (HK; 2.7.1.1), a possible target of phenothiazine, which had been widely used in the mid-20^th^ century as an anthelmintic^31^; (ii) Pantothenate kinase (PanK/CoA; 2.7.1.33), which is involved in CoA biosynthesis and possibly available at high levels in cells^32^ and (iii) 1-phosphatidylinositol 4-kinase (PI4K; 2.7.1.67), with several inhibitors under studies against malaria^33^ and *Cryptosporidium*^34^. The other classes were represented by one EC each, and included Fructose-bisphosphatase (FBPase; 3.1.3.11), a key enzyme in gluconeogenesis that has not been identified as a target in helminths; 3′,5′-cyclic-nucleotide phosphodiesterase (PDE; 3.1.4.17), inhibitors for which have been suggested to be good potential drugs for targeting parasitic nematodes due to their ability to disrupt the *C*. *elegans* life cycle and nematode-specific active binding sites^35^; and Leucyl aminopeptidase (LAP-1/cathepsin III; 3.4.11.1), a potential drug target in *Leishmania*^32^, known to be an excretory/secretory (ES) product and a modulator of host immune system^36^, and previously studied as a potential vaccine candidate for the liver fluke, *Fasciola hepatica*^37,38^.

**Table 1:** Characteristics of the top 10 prioritized nematode chokepoint enzymes.

EC	Chokepoint description	# of nematodes with chokepoint (/17)	# KEGG pathways containing EC	Sterile/Lethal *C*. *elegans* phenotype	Maximum 81HG score^17^ (/137; top 25% of genes)	*A*. *ceylanicum* Stage gene expression	
						Adult exp. percentile	Adult/Infective ratio percentile
1.1.1.27	L-lactate dehydrogenase	17	4	—	105	82.5%	86.3%
1.1.1.37	Malate dehydrogenase	17	4	Embryonic lethal	132	93.9%	81.3%
1.2.1.3	Aldehyde dehydrogenase (NAD+)	13	13	—	121	76.0%	62.1%
1.3.5.2	Dihydroorotate dehydrogenase (quinone)	12	1	—	120	60.3%	63.5%
2.7.1.1	Hexokinase	17	5	—	—	60.8%	91.6%
2.7.1.67	1-phosphatidylinositol 4-kinase	13	1	Sterile progeny	120	45.2%	40.1%
2.7.1.33	Pantothenate kinase	16	1	—	120	63.7%	51.9%
3.1.3.11	Fructose-bisphosphatase	15	3	—	105	70.1%	76.6%
3.1.4.17	3′,5′-cyclic-nucleotide phosphodiesterase	17	1	Sterile	123	62.9%	43.0%
3.4.11.1	Leucyl aminopeptidase	17	1	—	—	75.0%	62.1%

### First generation compound screening with parasitic nematodes in a phenotypic motility assay

To test the knowledge-based compound predictions for the 10 prioritized ECs (15 ChEMBL targets; 2,167 compound:target pairs; 1,581 drugs), 94 compounds were purchased and assayed in adult hookworm. Eleven (Table 2) had deleterious effects in hookworm (Motility Index, MI ≤0.75 for severe phenotype, 0.76 to 1.8 for moderate phenotype or had noticeable phenotype; 1.81 to 3, no phenotype) and were putatively targeting six of the 10 enzymes (success rate of 60% based on the target numbers), representing 3 different enzyme classes (2 dehydrogenases, 3 kinases, and a phosphodiesterase; Table 2). The 11 compounds causing severe or moderate phenotype in hookworm were also assayed in whipworm and seven were hits (i.e. had moderate to severe motility inhibition), hence classified as potential pan-intestinal compounds. Four of the seven compounds were potential pan-intestinal hits resulting in severe phenotypes (MI < 1.3) in both species, and two (**33** and **69**) had severe phenotype only in whipworm. One compound (**9**) showed moderate/noticeable phenotype in both species. Based on our analysis (Supplementary Fig. S1) the active compounds were linked to drug-like compounds shown to target MDH, PDE and PI4K/PI3K/mTOR in other species. The eight compounds (out of the 11 hits) associated with these enzymes (Table 2) were screened in non-intestinal nematode, adult stage of the filarial nematode *Brugia pahangi*, to examine their pan-phylum potential. Four (**15**, **47**, **58**, **59**) were effective at day 2, and 3 additional (**20**, **30**, **33**) at day 6 in *B*. *pahangi*, hence showing pan-Nematoda potential (motility inhibition of at least 80% at day 2 or day 5 was used as a cut-off for activity).

**Table 2:** Compound efficacies in a phenotypic whole worm assay. * - Miconazole tested at 10 uM, not at 30 uM. ** - Worms looked very sick).

Compound			Motility index (30uM, 48 hrs)		Motility inhibition % (30 uM, 48 hrs)	Target	
#	CHEMBL ID	Name or Formula	*A*. *ceylanicum*	*T*. *muris*	*B*. *pahangi (F)*	EC#	Descriptor
33	CHEMBL51483	Gossypol	1.5	**0**.**33**	22 **(83% at day 6)**	1.1.1.37	malate dehydrogenase
47	CHEMBL91	Miconazole	**0**.**75**	**0**	95*	1.1.1.37	malate dehydrogenase
58	CHEMBL808	Econazole	**0**.**25**	**0**	**99**	1.1.1.37	malate dehydrogenase
59	CHEMBL1221	Sulconazole	**0**	**0**	**99**	1.1.1.37	malate dehydrogenase
15	CHEMBL1256459	Torin 1	**0**.**5**	**0**.**6**	**100**	2.7.1.67	phosphatidyl-inositol kinase
20	CHEMBL1765602	PP121	1.58	2.42	7 **(100 at day 6)**	2.7.1.67	phosphatidyl-inositol kinase
30	CHEMBL202740	5-(2-(2-Methoxyphenylamino)thiazol-4-yl)-4-methylthiazol-2-amine	1.33	2	44 (**100 at day 6**)	3.1.4.17	phospho-diesterase
69	CHEMBL203287	4-({[3-cyano-6-(thiophen-2-yl)-4-(trifluoromethyl)pyridin-2-yl]sulfanyl}methyl)benzoic acid	1.61	**0**.**16**	0 (81 at day 6)	3.1.4.17	phospho-diesterase
87	CHEMBL1373924	N-(1,3-benzothiazol-2-yl)-3-methoxybenzamide	1.5	3	N/A	1.3.5.2	dihydroorotate dehydrogenase
9	CHEMBL474626	N-(4-methyl-1,3-thiazol-2-yl)-3 phenoxybenzamide	1.92**	1.67**	N/A	2.7.1.1	hexokinase
4	CHEMBL3410400	3-{4,6-dimethyl-3,7,8,10-tetraazatricyclo[7.4.0.02.7]trideca-1,3,5,8,10,12-hexaen-5-yl}-N-(4-acetamidophenyl)propanamide	1.67	2.75	N/A	2.7.1.33	pantothenate kinase

### Identification of second generation compounds with pan-phylum potential and screening with parasitic nematodes in a phenotypic motility assay

**Figure 2:**
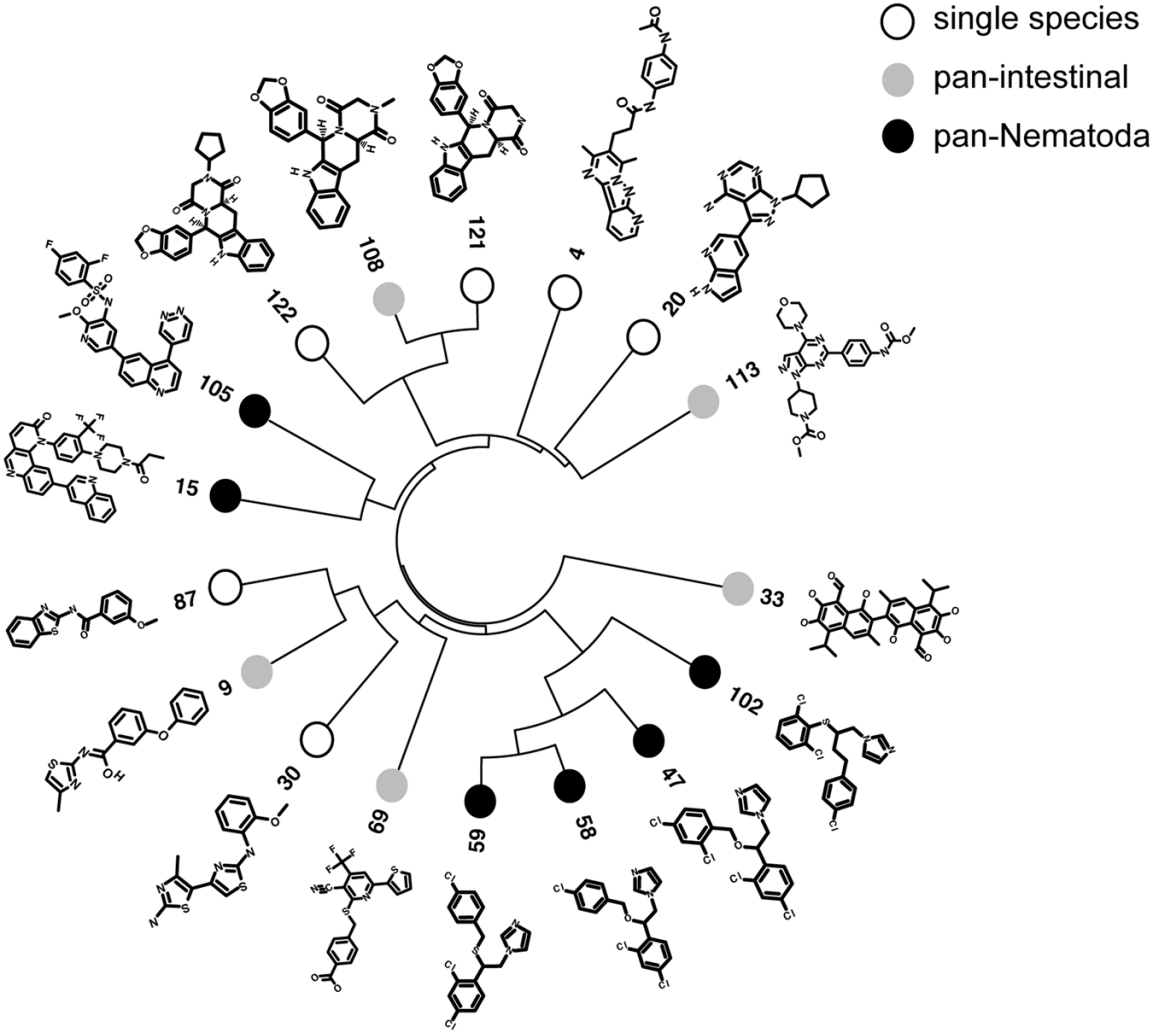
Structural similarity of the 17 active compounds, identified in the primary, secondary and tertiary screen of compounds for the identified targets. The clustering was based on (1 − Tanimoto similarity measure) as distance metric, calculated using ChemmineR^105^ package, and agglomerated using “complete linkage” method.

Based on the results from the 94 first generation compounds we identified three effective target classes - MDH, PDE and PI4K/PI3K/mTOR - and expanded the compound list by structural similarity and pharmacophore searches, resulting in identification and screening of 26 second generation compounds (Supplementary Table S4). This compound expansion and screening in the intestinal and filarial species led to the identification of four (**102**, **105**, **108**, **113**) further active potential pan-intestinal molecules, of which two (**102** and **105**) have pan-Phylum molecules (Fig. 2, Supplementary Table S4) in this series. The putative target-associated compounds that were not active in this assay, were not necessarily structurally related to the first-generation inhibitors.

Overall the screening of the first and second generation compounds resulted in identification of twelve active compounds that putatively target three homologous therapeutic targets - MDH, PI3K/mTOR and PDEs (Table 3) in at least one parasitic species.

**Table 3:** First and second generation active compounds and their targets in literature.

Comp. #	CHEMBL ID	Common name	Literature target
**33**	CHEMBL51483	Gossypol	LDH^49^, MD^50^, BLC2L1^51^, MCL-1^52^, APE1^53^
**47**	CHEMBL91	Miconazole	14alpha-demethylase; certain ion channels and receptors^54–57^, MDH^48^
**58**	CHEMBL808	Econazole	
**59**	CHEMBL1221	Sulconazole	
**102**	CHEMBL1200398	Butaconazole Nitrate	
**15**	CHEMBL1256459	Torin 1	mTOR/PI3K/other kinases^59^
**105**	CHEMBL1236962	Omipalisib	PI3K/mTOR^60^
**113**	CHMEBL561708	WYE-354	mTOR/PI3K^61^
**30**	CHEMBL202740	—	PDE1/PDE5^66^
**69**	CHEMBL203287	—	PDE1/PDE5^66^
**108**	CHEMBL779	Tadalafil	PDE5/PDE11^67^

### Screening of known azoles

Four of our active first-generation compounds were prioritized based on their known activities against MDH^39,40^ although they have numerous discrete canonical targets (Table 3). Gossypol is a polyphenol known to target lactate dehydrogenase^41,42^ and BCL2 family proteins involved in apoptosis and APE-1, which participates in DNA damage repair^43–45^. The other three were azoles (Miconazole, Econazole and Sulconazole), which are imidazole derivatives known to be antifungal agents. They inhibit P-gp and multiple CYP proteins. One of the CYP proteins (14 alpha-demethylase) is thought to be the main target that leads to antifungal activity by inhibiting synthesis of ergosterol, an important steroid necessary for permeability of the fungal cellular membrane^46,47^. These azoles also display other activities, including inhibition of certain ion channels and receptors^48,49^. We included Butaconazole, also an azole, among our second-generation compounds based on the successful motility inhibition shown by these three azoles. Like the other three azoles, Butaconazole is also predicted to inhibit 14 alpha-demethylase^48^. In our *in vitro* assays, Sulconazole had IC_50_s of 11.8 µM against the whipworm *T*. *muris* and 4.6 µM against the filarial worm *B*. *pahangi*.

### Screening of known PI3K, PI4K lipid kinases and mTOR inhibitors

Our discovery that the mixed mTOR/PI3K/PI4K inhibitor Torin1 (**15**) showed efficacy in inhibiting worm motility in both hookworms and whipworms prompted us to test other known and more selective inhibitors of each of these kinases. The high prioritization score of **15** was based on its high potency as an inhibitor of mTOR with an IC_50_ of 2 nM and 10 nM in the protein complexes mTORC1 and mTORC2, respectively^50^. In the same study, it exhibited 1000-fold selectivity of mTOR over PI3K in mammalian cells. In our assays, the IC_50_ for **15** was 13.3 µM in the whipworm *T*. *muris* and 3.9 µM in the filarial worm *B*. *pahangi*. Intriguingly, the structurally related inhibitor Torin-2 (**23**) did not exhibit similar activity, nor did the other PI4K kinase inhibitors (**95**, **106**, **109**, **116**, and **120**) (Supplementary Table S4). Therefore, we identified and evaluated additional mTOR inhibitors and found that Omipalisib^51^ (**105**) and WYE-354^52^ (**113**) showed equivalent or better activity against both these nematodes (Fig. 2). Interestingly, **105** showed better activity than **113**, a more selective inhibitor of mTOR. Because the structurally related inhibitor Torin-2 did not exhibit activity and Omipalisib did, we screened more selective PI3K inhibitors and additional mTOR inhibitors to help better indicate the mechanism of action (Supplementary Fig. S3). The three additional drugs were: 1. CC-223 (**124**): a potent, selective, and orally bioavailable mTOR inhibitor with IC_50_ of 16 nM, >200-fold selectivity over the related PI3K-α^53^, 2. MLN1117 (Serabelisib; **125**): selective p110α (a PI3K) inhibitor with an IC_50_ of 15 nM^54^, and 3. XL388 (**126**): a highly potent ATP-competitive mTOR inhibitor with an IC_50_ of 9.9 nM^55^ which simultaneously inhibits both mTORC1 and mTORC2. None of the three showed significant activity. Dual inhibitors such as PF-04979064 exist^56^ and testing them can provide further clarification.

### Screening of known PDE inhibitors

**Figure 3:**
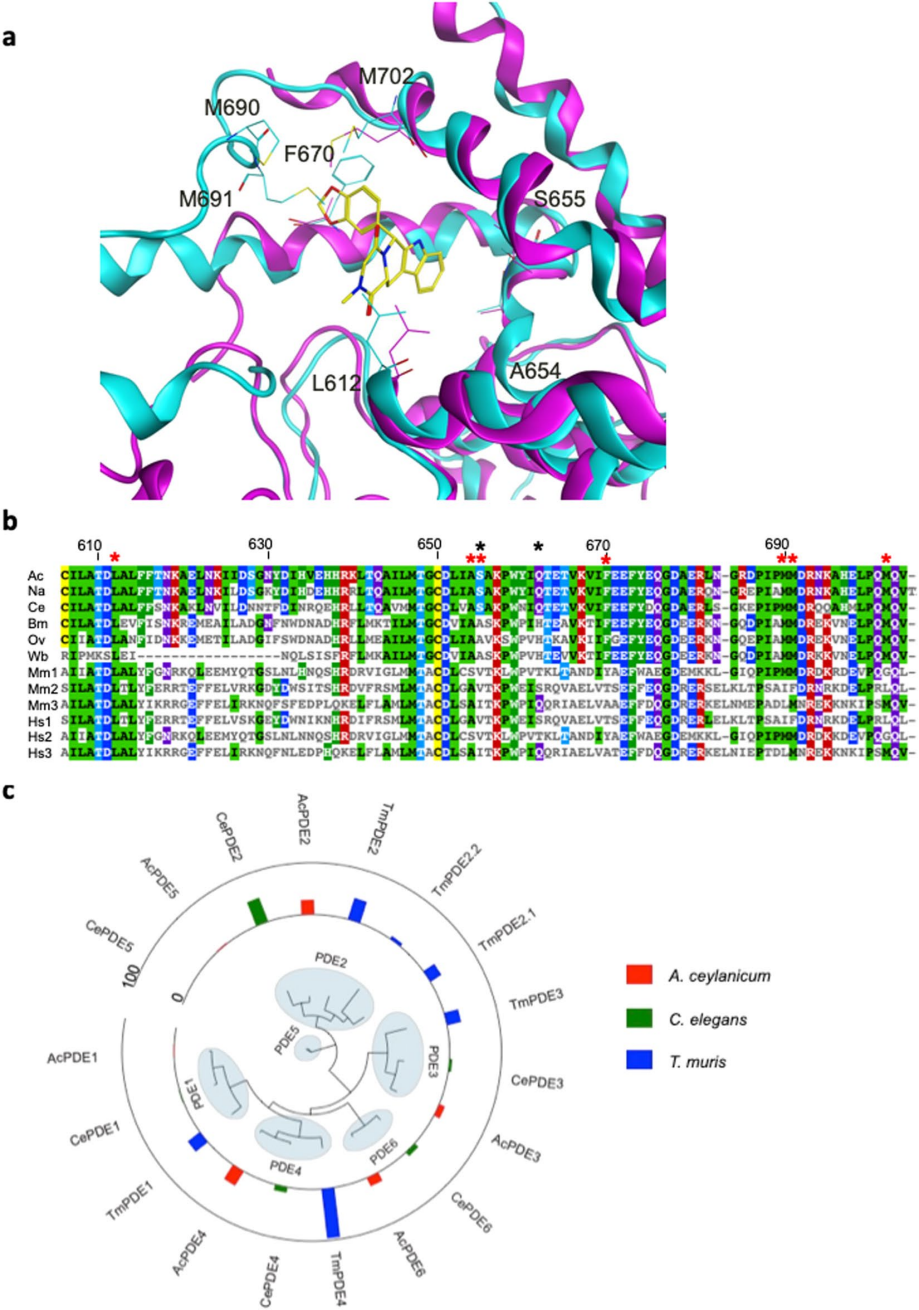
Characterizing PDE as a potential target (**a**) Tadalafil docked to human (magenta) and *A*. *ceylanicum* (cyan) PDE5 homology model, showing the interacting residues. (**b**) Sequence alignment of PDE5 homologs from 6 worms (Ac = *A*. *ceylanicum*, Na = *N*. *americanus*, Ce = *C*. *elegans*, Bm = *B*. *malayi*, Ov = *O*. *volvulus*, Wb = *W*. *bancrofti*) and 3 paralogs each from the hosts human (Hs) and mouse (Mm). No homolog was identified in *T*. *muris*. The region shown here is selected to illustrate that most of the interacting residues from panel A are conserved among nematodes and divergent from host species (indicated with red asterisks). Residues at or near the active site that are different between filarial and non-filarial worms are highlighted with a black asterisk. The residue numbers are based on *A*. *ceylanicum* PDE5. The residues matching the aligned residue in *A*. *ceylanicum* are colored, with the color depicting the residue type. (**c**) A maximum-likelihood phylogenetic tree based on sequence similarity of PDEs present in *A*. *ceylanicum*, *T*. *muris* and *C*. *elegans*, and their expression levels in adult stages (FPKM – fragments per kilobase per million mapped reads - plotted as bars in the outer track). PDE family clusters are indicated using shaded ellipses. The sequences were aligned using MAFFT^106^. The phylogenetic tree is estimated using PhyML 3.0^107^ and the node support values are calculated using “aLRT SH-like” option. All node support values are >=0.79.

We identified a weak PDE1/PDE5 inhibitor (**69) (**IC_50_ 1.9 µM/0.7 µM respectively^57^) in the worm assays and, subsequently, a more potent inhibitor Tadalafil^58^ (**108**) among the second-generation compounds. The additional seven PDE inhibitors tested (Supplementary Table S4) are from several chemical series, which encompass varied PDE family selectivity profiles. Intriguingly, Tadalafil was the most active PDE inhibitor, while other classes including Sildenafil (**110**) and Vardenafil (**89**) did not show activity against the representative hookworm or whipworm (Supplementary Table S4). The compounds we tested so far are largely selective for PDE5, PDE1 and PDE6 although our lead Tadalafil also shows significant activity for PDE11 (IC_50_ 15 nM)^59^, for which a direct ortholog is not found in nematodes even though PDE5 is the most closely related nematode enzyme to mammalian PDE11s (Supplementary Fig. S4). Tadalafil is a PDE5 inhibitor with IC_50_ of 9.4 nM and was at least 1000 times more selective for PDE5 than most of the other families of PDEs, with the exception of PDE11^58^. Subsequent screening of BC-11-38 (**123**), a selective PDE11 inhibitor^60^, showed no activity against whipworm (Supplementary Table S4). To further explore the Tadalafil scaffold, and to test the hypothesis that we are hitting PDE (and that worm PDE is similar to human), Tadalafil and *N*-cyclopropyl Tadalafil were docked into *A*. *ceylanicum* PDE5 homology model (Tadalafil docking shown in Fig. 3a). Acknowledging that uptake may be differentiated, we chose two compounds N-Desmethyl Tadalafil (**121**) and N-Desmethyl N-cyclopentyl Tadalafil (**122**) with varying ClogP (1.864 and 3.7944, respectively) for screening in whipworm. Both compounds showed activity (Supplementary Table S4). We note that most of the residues important for interactions with Tadalafil are conserved among Nematoda, but are divergent from the hosts (Fig. 3b). Additional studies are needed to determine the target, in light of the difference in PDE geneset among species (e.g. lack of PDE5 and two additional PDE2 in whipworm; Supplementary Fig. S4) and the PDE expression profiles in adult worms (Fig. 3c; Supplementary Table S5). Both **69** and **108** were not active against the filarial nematode *B*. *pahangi*. Taxonomically restricted residues in PDE5 exist among intestinal and filarial nematodes (Fig. 3c) and further focused studies are needed to determine their role in the observed differential activity.

### Putative nematode targets are involved in diverse metabolic pathways

The compounds that caused the most severe phenotypes putatively target three nematode enzymes that are involved in six metabolic pathways. PIK (EC2.7.1.67) and PDE (EC3.1.4.17) are involved in inositol phosphate metabolism and purine metabolism, respectively and MDH (EC1.1.1.37) is involved in four other pathways (described below).

PIK is a transferase that transfers phosphorus-containing groups (phosphotransferases with an alcohol group as acceptor) which is involved in carbohydrate metabolism (inositol phosphate metabolism). A previous study has identified another enzyme in the inositol phosphate metabolism pathway, inositol polyphosphate multikinase (IPMK, EC2.7.1.151), as a druggable and lethal target in kinetoplastid parasites^61^, showing vulnerability of this pathway in other parasites.

PDEs are hydrolases that cleave ester bonds (phosphoric-diester hydrolases) and are involved in purine nucleotide metabolism. Despite the widespread notion that nematodes cannot synthesize purines^62^, complete or near-complete purine synthesis pathways were recently found in most members of clades I, IIIb (Ascaridomorpha) and V^12^. This critical function highlights purine metabolism as a promising drug target, as evidenced here by the efficacy of targeting PDE; notably, inhibitors of PDE have been suggested as promising potential drugs against parasitic nematodes given their ability to disrupt the *C*. *elegans* life cycle and existence of nematode-specific active binding sites^35^. Ten other high priority chokepoints are part of the purine metabolism pathway (Supplementary Table S6). For example, previously we have prioritized pyruvate kinase (EC 2.7.1.40), an enzyme upstream of PDE, as a top promising conserved chokepoint drug target,^1^ and due to its critical function and diversification in the active site compared to the human host it has also been identified as a promising drug target for malaria^63^ and *Staphylococcus aureus*^64^. Pyruvate kinase is also a chokepoint in pyruvate metabolism pathway.

MDH is an oxidoreductase that acts on the CH-OH group of donors with NAD+ as acceptor. It is involved in three carbohydrate metabolism pathways (pyruvate, glyoxylate/dicarboxylate, and TCA cycle) and also in amino acid metabolism (cysteine and methionine). It is essential to energy production through both aerobic and anerobic pathways, making it essential for parasite’s survival even in very diverse niches. Some anthelmintics appear to target MDH activity^65^ and it was originally thought to be the possible target of benzimidazole anthelmintics, with Mebendazole shown to inhibit MDH activity *in vitro*^66^. Multiple enzymes from carbohydrate metbolism and glycolytic pathways, including MDH, have been studied for their potential as anthelmintic targets^67–69^. There are 7 other enzymes among our top 50 chokepoints list (Supplementary Table S2) that are also part of pathways that involve MDH (4 in pyruvate metabolism and 4 in cysteine/methionine metabolism; one enzyme - EC 1.1.1.27 - is involved in both these pathways). Many of these have been suggested and explored as potential anti-parasitic, antifungal or anthelmintic drugs^63,70–80^.

### *In vivo* efficacy of lead compounds

**Figure 4:**
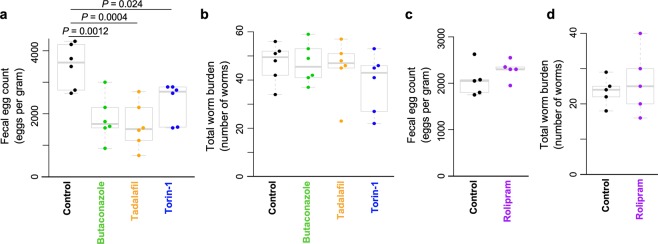
Butaconazole, Tadalafil, Torin-1 reduced fecal egg count but not total worm load and rolipram did not reduce either fecal egg count or total worm load. (**a**) Treatment with each of the three drugs significantly reduced fecal egg count in Syrian hamsters infected with the hookworm *A*. *ceylanicum* compared with untreated control animals. (**b**) The fecal egg count reduction was not accompanied by a reduction of worm load, which was not statistically significant between control and treated animals. No reduction in fecal egg count (**c**) or worm burden (**d**) in Syrian hamsters infected with the hookworm *A*. *ceylanicum* when treated with rolipram compared to control animals.

To ascertain whether the newly discovered classes of pan-nematode chokepoint inhibitors possessed therapeutic utility in parasitic nematodes, Syrian hamsters (*Mesocricetus auratus*) infected with *A*. *ceylanicum* were treated with representative drugs from each of the three target classes at 100 mg/kg *per os*, based on previously reported effective dosage of currently used major anthelmintics^81^. Three (Butaconazole, Tadalafil and Torin-1) of the four tested compounds that were active *in vitro* had significant and noteworthy impact on eggs per gram of feces indicating treatment affected parasite fecundity, although the treatment had no marked effect on worm burden (Fig. 4a,b). Rolipram, a drug that was not active *in vitro*, did not have an impact on eggs per gram or on worm burden (Fig. 4c,d).

In conclusion, omics-driven knowledge-based identification of drug targets is a powerful approach to identify targets essential for organisms survival. Undertaking this approach we were able to prioritize 10 of the 186 identified conserved chokepoint enzymes and undertake a target class repurposing approach to test and identify drugs with broad spectrum anthelmintic activity. First, we tested 94 compounds using an *in vitro* phenotypic assay, and identified 11 actives. Based on the findings and SAR, we tested 32 additional compounds, identifying 6 more actives. Among the 17 actives 5 are potential pan-intestinal and 6 are potential pan-Nematoda drugs. The active compounds target three different target classes. Using representative drugs from each target class, we demonstrated efficacy in hamsters infected with hookworm, as characterized by negative effects on parasite fecundity. While the leading scaffolds require optimization, the study identified target classes and essential pathways for parasitic nematode survival.

## Materials and Methods

### Enzyme annotation, nematode chokepoints identification and taxonomic classification

High confidence annotation of nematode metabolic enzymes was performed as described^12^. In short, the multi-step process assigns Enzyme Commission (EC) numbers to sequences in proteomes of interest. Multiple EC-annotation approaches that were employed included KAAS^82^, PRIAM^83^, DETECTv2^84^ and BRENDA^85^. For species with good quality draft genomes (including the representative intestinal species, *T*. *muris* and *A*. *ceylanicum*), the pathway hole-filling algorithm of the Pathway Tools package^86^ was used to identify additional metabolic enzymes.

### Identification and prioritization of nematode chokepoint targets and drug

For nematode chokepoint EC prioritization we first quantified pan-Nematoda chokepoint conservation by performing chokepoint analysis on 17 species spanning the phylum (Supplementary Fig. S1 and Supplementary Table S1): *Ancylostoma ceylanicum*^87^, *Ancylostoma duodenale*^12^, *Ascaris lumbricoides*^12^, *Ascaris suum*^88^, *Brugia malayi*^89^, *Dirofilaria immitis*^90^, *Heligmosomoides bakeri*^12^, *Loa loa*^91^, *Necator americanus*^92^, *Nippostrongylus brasiliensis*^12^, *Onchocerca volvulus*^87^, *Strongyloides ratti*^93^, *Strongyloides stercoralis*^93^, *Trichuris muris*^94^, *Trichuris suis*^12^, *Trichuris trichiura*^94^, and *Wuchereria bancrofti*^12^. Second, chokepoint ECs were assigned a score with a maximum value of 6, based on five different criteria: (i) ECs with higher levels of pan-Nematoda conservation received a higher score, calculated using (2× [Number of species/17]), with a maximum value of 2; (ii) Chokepoints annotated to multiple KEGG^95^ pathways received a higher score, calculated using (number of pathways/4), to a maximum value of 1; (iii) If the *C*. *elegans* ortholog has a “lethal” or “sterile” phenotype annotated by WormBase^96^, it received 1 point; (iv) The chokepoints among the top 25% targets scored in the “Comparative genomics of the major parasitic worms” study (International Helminth Genomes Consortium^12^) received a score calculated by (score/137), maximum value 1. This score was based on multiple factors, including lack of human homolog, presence of a *C*. *elegans* or *Drosophila melanogaster* homolog with a deleterious phenotype, expression in key life cycle stages, widespread conservation among helminths, lack of paralogs, presence of a related structure in PDB, etc. The top quartile among all the scored proteins were given a positive score by us, as they represented potentially good anthelmintic targets; and (v) RNA-Seq-based gene expression levels were used to prioritize ECs based on data from *A*. *ceylanicum* (0.5 x the percentile of the EC’s adult-stage expression compared to all other genes) and *T*. *muris* (0.5 x the percentile of the EC’s [adult stage/free-living larval stages] fold change value compared to all other genes), with a maximum value of 1. The distribution of all of the contributing scores and the final scores are shown in Supplementary Fig. S2.

For the ChEMBL target:drug pair prioritization, ChEMBL^19^ targets were annotated among the two target species (minimum 40% identity across 75% of the protein’s length, *P* ≤ 10^−10^) and subsequently ChEMBL was used to match drugs, identifying target:drug pairs with a pChEMBL score ≥5. The drugs that had a “Quantitative Estimate of Druglikeness” (weighted QED) score available and were associated with one of the prioritized 186 nematode metabolic chokepoints were identified. The final ChEMBL target:drug scores were calculated by multiplying (i) the pChEMBL drug:target affinity scores, scaled between 0.5 and 1, and (ii) the weighted QED scores, scaled between 0 and 1. Final prioritization score was calculated by multiplying the final nematode chokepoint EC score and the final ChEMBL target:drug score (Supplementary Fig. S1). Last, significant sequence similarity to either a human or a mouse protein with a structure in PDB (sequence match with an E value < 10^−10^) was required for a drug:target pair to be included for further consideration.

For each of the 50 target ECs with available PDB structure, the nematode sequence was aligned with that of the PDB structure using MOE (Chemical Computing Group, Inc.). A two-step visual inspection of the ligand binding site ranked targets based on (i) identification of a ‘druggable’ binding site^97^, with compound bound where possible, and (ii) protein sequence differences between the nematode and host sequences in the region of the binding site (this step was preceeded by manual improvement of the nematode gene models guided by transcriptional evidence). This process resulted in 10 priority targets.

### Identification of small molecule inhibitors

First generation compounds: For the 10 prioritized EC targets, we identified all reported active compounds in ChEMBL. These were assessed for drug-likeness; PAINS^98^ were eliminated. Of these, 94 compounds were available for purchase. Both hookworm and whipworm assays were carried out for an initial batch of 30 compounds. Based on the results of these, it was decided to employ a stepwise approach for the remaining first generation compounds i.e. hookworm, then whipworm, then *Brugia*, contingent on activity in the previous step(s). Second generation compounds: Based on the results from 94 first generation compounds, five proteins were reasoned as the potential biologically relevant targets for our active hits and thus more readily commercially available compounds known to target each of these were included (n = 26). These were five PI4K/PI3K inhibitors, nine mTOR inhibitors, five PDE inhibitors, two potential malate dehydrogenase inhibitors, and five Econazole analogs. The mTOR inhibitors were included based on CHEMBL1256459 (Torin1) - a potent inhibitor of mTORC1/2. Third generation compounds: Based on the results from the 26 second generation compound, additional compounds were identified and screened (n = 6). These included three PDE inhibitors, two mTOR and one PI3K (p110α) inhibitor.

### Compound screening with adult stage of intestinal nematode *Ancylostoma ceylanicum*

*In vitro* assays were carried out as described^81^. Briefly, young adult *A*. *ceylanicum* were harvested from the small intestine of infected hamsters on day 11 post-inoculation (p.i.) and washed three times using prewarmed medium (RPMI1640, 100 U penicillin, 100 μg/ml streptomycin, 10 μg/ml amphotericin). Three worms were manually picked into each well of the 96-well, 4 wells/test drug, containing 99 μl of medium. 1 μl of 3 mM (100% DMSO) drugs were transferred into the corresponding well giving a final concentration of 30 µM (1% DMSO) and 100 µl final volume. Assay plates were incubated for 48 hours at 37 °C and 5% CO_2_. Drug activity was determined every 24 hours using the standard motility index. Motility index of 3 were given to vigorous worms, 2 for motile worms, 1 for motile after stimulation by touching and 0 for dead worms. Pyrantel pamoate 30 µM was used as positive control prepared exactly as the test drugs, while 1% DMSO was used as negative control.

### Compound screening with adult stage of intestinal nematode *Trichuris muris*

*In vitro* assays were carried out as described^81^. Briefly, *T*. *muris* adult whipworms were harvested from the cecum and large intestines of infected STAT6 −/− mice. Three worms were manually picked into each well of a 24-well plate, 2 wells/ test drug, containing 990 µl. 10 µl of 3 mM (100% DMSO) drugs were transferred into the corresponding well giving a final concentration of 30 µM (1% DMSO) and a final volume of 1 ml of medium. Test plates were incubated for 48 hours at 37 °C and 5% CO_2_. Drug activity was assayed using the standard motility index as per hookworm adults. Levamisole 30 µM was used as positive control prepared exactly as the test drugs, while 1% DMSO was used as negative control. IC_50_ values at 48 hours were established using a non-linear regression with Prism 7 (Graphpad, La Jolla, CA). Only IC_50_s displaying R^2^ values ≥ 0.7 are reported.

### Compound screening with adult stage of the filarial nematode *Brugia pahangi*

Adult female *B*. *pahangi* worms were obtained from Dr. Brenda Beerntsen, University of Missouri, Columbia, MO. Individual females were placed in each well of a 24-well plate in culture medium (RPMI-1640 with 25 mM HEPES, 2.0 g/L NaHCO3, 5% heat inactivated FBS, and 1X Antibiotic/Antimycotic solution.

Initial screening of the compounds was performed at 10 μM for Miconazole, Econazole, and Sulconazole. Gossypol, Torin2, PP121, and Torin1 were initially screened at 30 μM and 10 μM. Four worms were used as replicates at each concentration in 500 uL of media. Worms treated only with 1% DMSO served as the negative control. The cultures were maintained at 37 °C, 5% CO_2_ incubator for seven days - the duration of the assay.

The Worminator, a visual imaging software, was used to determine the effect of each treatment on worm motility^99^. Movements of individual worms were calculated by determining the number of pixels displaced per second per well. Worm movements were averaged over the time of recording (60 seconds) and mean movement units (MMUs) were determined for individual worms. Percent inhibition of motility was calculated by dividing the MMUs of the treated worms by the average MMUs of the 1% DMSO treated control worms, subtracting the value from 1.0, flooring the values to zero and multiplying by 100%. Videos of the worms during the assay were recorded on days 0, 1, 2, 3, and 6 of the incubation. Compounds showing ≥ 75% inhibition of motility at either concentration on day 3 of the assay were investigated further to determine IC_50_s. Worms were treated in the same fashion as above and motility was measured each day for three days using the Worminator. IC_50_ values on day 3 (Torin 1 and Sulconazole) were established using a non-linear regression curve fit with Prism 7 (Graphpad, La Jolla, CA). All IC_50_s with R^2^ values ≥ 0.7 are reported.

### *In vivo A*. *ceylanicum* assay

Assays were conducted essentially as previously^81,100^. Briefly, 5 weeks old golden Syrian hamsters were infected with 120 third-stage larvae of *A*. *ceylanicum*. Infected rodents were grouped based on the fecal egg count on day 18 p.i. Each group had five hamsters. Drugs were delivered in water suspension after completely dissolved in 100% DMSO giving a final dose of 100 mg/kg and 0.1% DMSO volume to body weight. Control group was dosed only with water and 0.1% DMSO. Fecal samples were collected 21 days p.i. for fecal egg counts. On day 22 p.i., the hamsters were euthanized, and the small intestine was opened longitudinally. Adult parasites within the intestine were counted under a dissecting microscope.

A 100 mg/kg dosing for *in vivo* experiments was chosen based on similar studies from other groups as maximum single dosing for anthelmintic compounds^81,101–103^. For comparison, with current clinically approved anthelmintics high efficacy is seen with doses at 5–10 mg/kg^103,104^.

### Ethics statement

All animal experiments were carried out under protocols approved by University of Massachusetts Medical School (UMMS; A-2483 and A-2484) Institutional Animal Care and Use Committees (IACUC). All housing and care of laboratory animals conformed to the National Institutes of Health (NIH) Guide for the Care and Use of Laboratory Animals in Research (see 18-F22) and all requirements and all regulations issued by the United States Department of Agriculture (USDA), including regulations implementing the Animal Welfare Act (P.L. 89–544) as amended (see 18-F23). Euthanasia was accomplished by CO_2_ asphyxiation, followed by bilateral pneumothorax.

## Supplementary information

Supplementary Information

Supplementary Tables

## Data Availability

The datasets generated during and/or analysed during the current study are available from the corresponding author on reasonable request.
