# Supplementary material for: Identification of small molecule enzyme inhibitors as broad-spectrum anthelmintics
Source: Sci Rep. 2019 Jun 24;9:9085. doi: 10.1038/s41598-019-45548-7 (PMC6591293; doi:10.1038/s41598-019-45548-7)
Supplement: Supplementary file 1 — Supplementary Information [file 41598_2019_45548_MOESM1_ESM.pdf]

Supplementary Information

for

Identification of small molecule enzyme inhibitors as broad-spectrum anthelmintics

Rahul Tyagi[1#], Mostafa A. Elfawal[2#], Scott A. Wildman[3], Jon Helander[4], Christina A. Bulman[5], Judy Sakanari[5], Bruce A. Rosa[1], Paul J. Brindley[6], James W. Janetka[4], Raffi V. Aroian[2] and Makedonka Mitreva[1,7*]

[1]McDonnell Genome Institute, Washington University School of Medicine, 4444 Forest Park Ave, St. Louis, Missouri 63108, USA

[2]University of Massachusetts Medical School, Suite 219 Biotech 2, 373 Plantation St., Worcester, Massachusetts 01605, USA

[3]UW Carbone Cancer Center, School of Medicine and Public Health, University of Wisconsin-Madison, 1111 Highland Ave., Madison, Wisconsin 53792, USA

[4]Department of Biochemistry and Molecular Biophysics, Washington University School of Medicine, 660 S. Euclid Ave., Box 8231, St. Louis, Missouri 63110, USA

[5]Department of Pharmaceutical Chemistry, University of California San Francisco, 1700 4th St, San Francisco, California 94158, USA

[6]Department of Microbiology, Immunology & Tropical Medicine, and Research Center for Neglected Diseases of Poverty, School of Medicine and Health Sciences, George Washington University, Ross Hall, Room 521, 2300 I Street, NW, Washington, DC 20037, USA

[7]Division of Infectious Diseases, Department of Medicine, Washington University School of Medicine, 4523 Clayton Ave., CB 8051, St. Louis MO, 63110, USA

[#]Equal contribution

*Corresponding Author (email: mmitreva@wustl.edu).

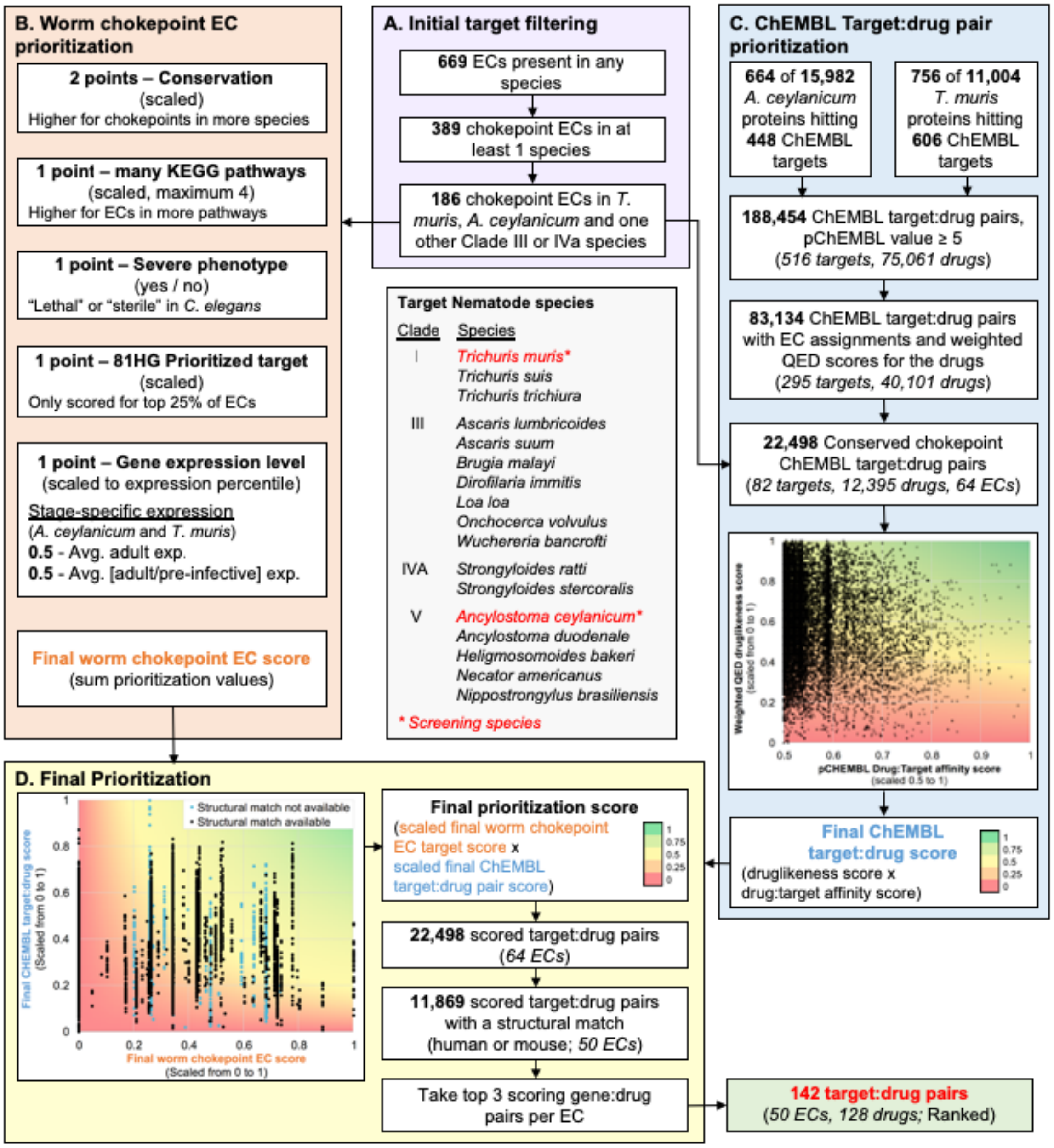

Supplementary Figure S1. Detailed flowchart outlining the overall analysis pipeline.

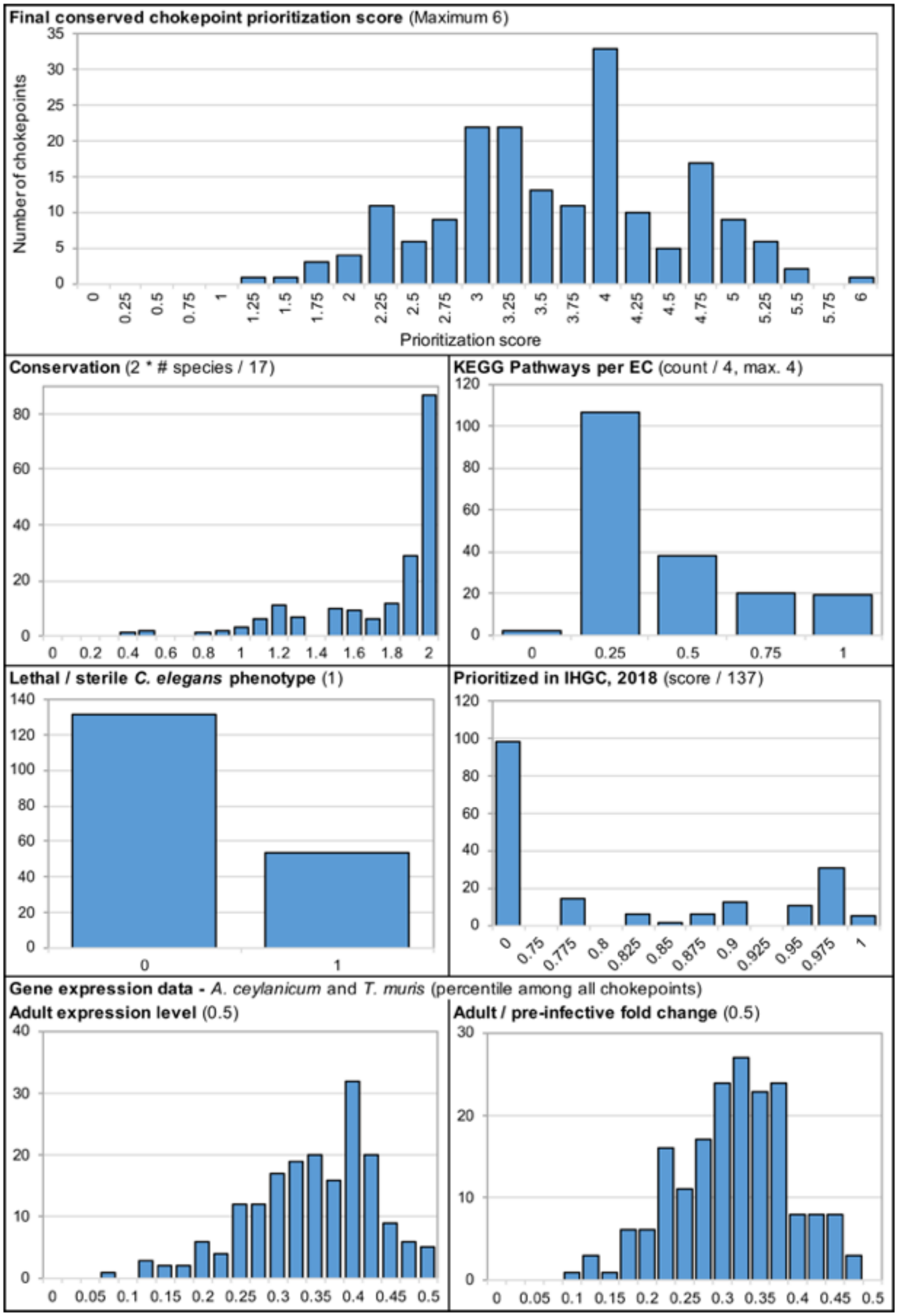

Supplementary Figure S2. The distribution of prioritization scores for the 186 scored chokepoints that were identified among *T. muris, A. ceylanicum* and one other Clade III or IVa nematode species.

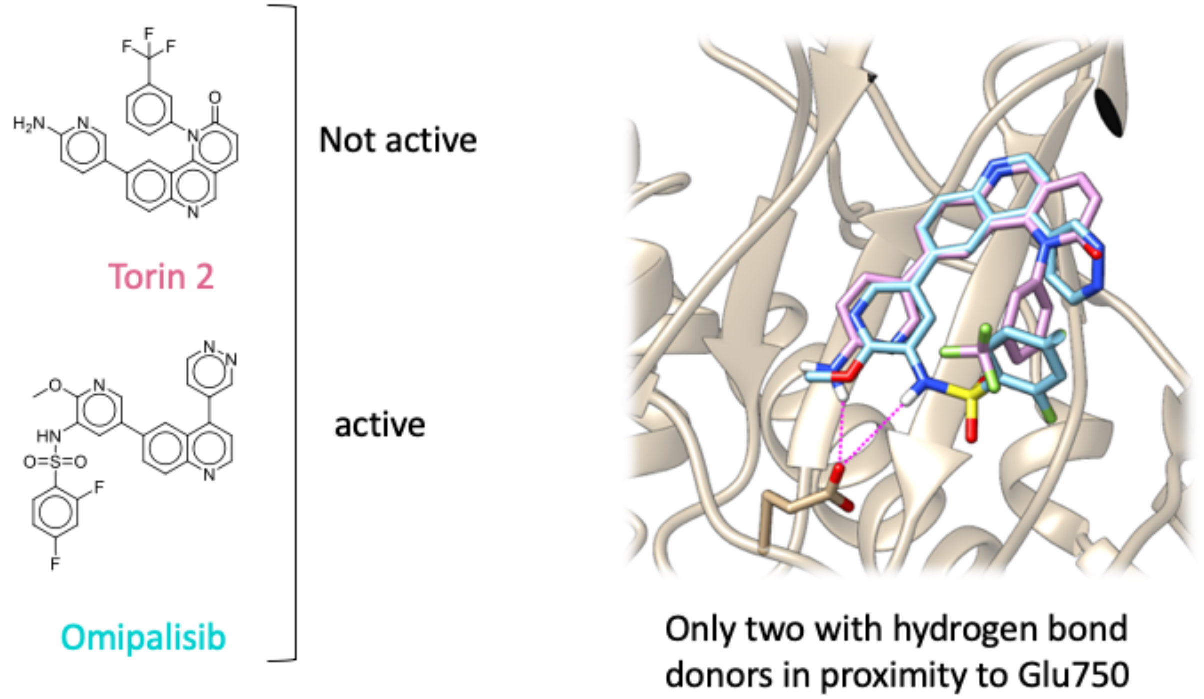

Supplementary Figure S3. Binding model of inactive Torin2 (pink) and active Omipalisib (cyan) to mTOR.

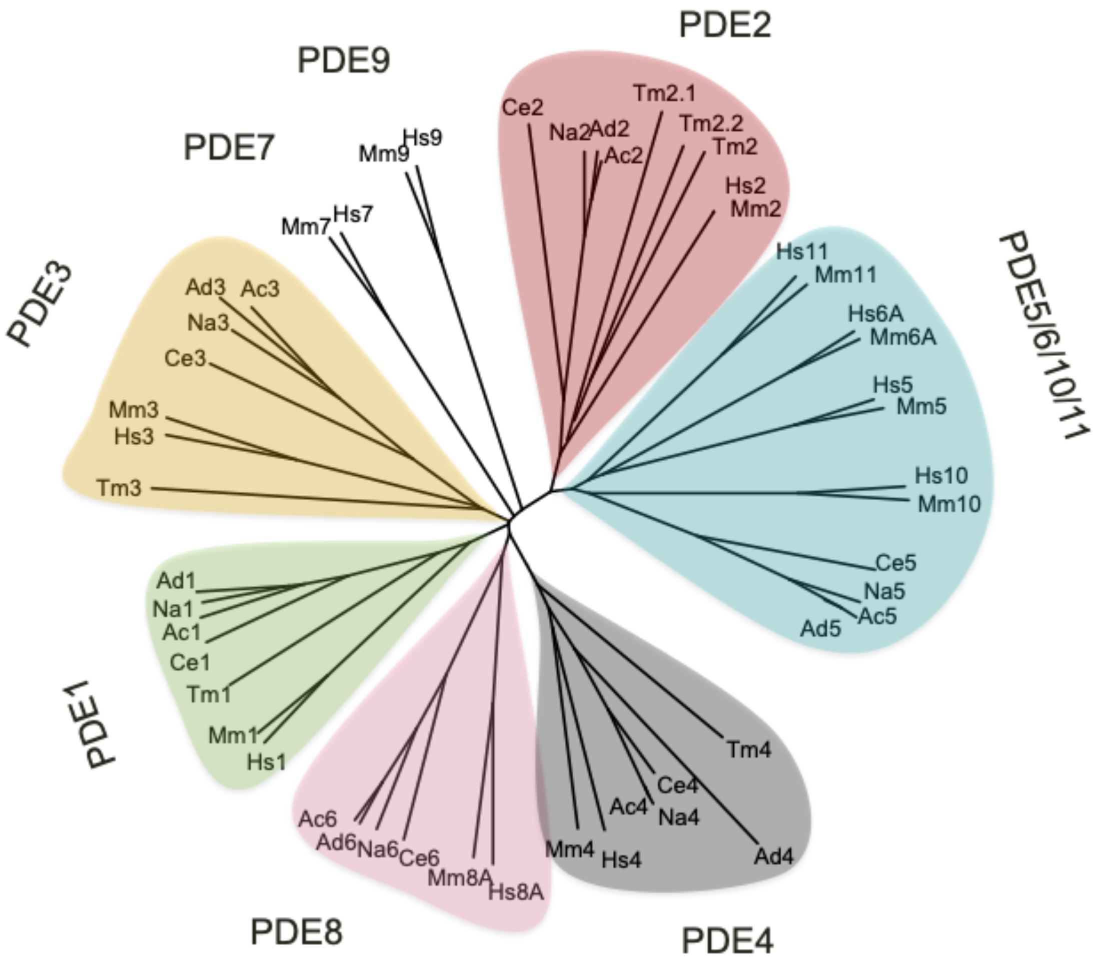

Supplementary Figure S4. Phylogenetic relationship of PDE genes in hookworm (Ac, *A. ceylanicum*; Ad, *A. duodenale*; Na, *N. americanus*), whipworm (Tm, *T. muris*), *C. elegans* (Ce), and hosts human (Hs) and mouse (Mm). The cluster names are based on the human homologs, which may not necessarily be same as the worm homologs (e.g. PDE6 in worms are more closely related to Hs8, than Hs6). Tm2.1 and Tm2.2 are two candidate PDE2 paralogs in *T. muris*.

Supplementary Tables

Supplementary Table S1. All identified chokepoint enzymes for each of the 17 species inclued in the analysis and the corresponding genes.

Supplementary Table S2. The 50 nematode chokepoint enzymes with hits to available human/mouse homolog structures on PDB.

Supplementary Table S3. PDB hits to the nematode chokepoint enzymes.

Supplementary Table S4. Whole worm phenotypic assay results of the *in vitro* screening of various commercially available compounds on multiple nematode worms.

Supplementary Table S5. Late larval stage and Adult stage expression in *A. ceylanicum* and *T. muris* of genes corresponding to the putative chokepoint targets of compounds that showed severe phenotypes.

Supplementary Table S6. Additional high priority chokepoints in pathways of interest.
